# Novel Electrospun Composite Membranes Based on Polyhydroxybutyrate and Poly(vinyl formate) Loaded with Protonated Montmorillonite for Organic Dye Removal: Kinetic and Isotherm Studies

**DOI:** 10.3390/membranes13060582

**Published:** 2023-06-03

**Authors:** Hristo Penchev, Ahmed E. Abdelhamid, Eman A. Ali, Dessislava Budurova, Georgy Grancharov, Filip Ublekov, Neli Koseva, Katerina Zaharieva, Ahmed A. El-Sayed, Ahmed M. Khalil

**Affiliations:** 1 Institute of Polymers, Bulgarian Academy of Sciences, Acad. G. Bonchev 103A, 1113 Sofia, Bulgaria; dbudurova@gmail.com (D.B.); granchar@polymer.bas.bg (G.G.); ublekov.philip@gmail.com (F.U.); koseva@polymer.bas.bg (N.K.); 2 Polymers and Pigments Department, National Research Centre, El-Buhouth Str., Giza 12622, Egypt; ahmednrc10@gmail.com (A.E.A.); emabal2003@yahoo.com (E.A.A.); 3 Institute of Mineralogy and Crystallography Acad. I. Kostov, Bulgarian Academy of Sciences, Acad. G. Bonchev 107, 1113 Sofia, Bulgaria; zaharieva@imc.bas.bg; 4 Photochemistry Department, National Research Centre, El-Buhouth Str., Giza 12622, Egypt; ahmedcheme4@yahoo.com

**Keywords:** electrospinning, nanocomposite fibers, polyhyrdoxybuterate, poly(vinyl formate), formic acid, protonated montmorillonite, dye removal, methylene blue, Congo red

## Abstract

The use of biodegradable polyesters derived from green sources and their combination with natural abundantly layered aluminosilicate clay, e.g., natural montmorillonite, meets the requirements for the development of new sustainable, disposable, and biodegradable organic dye sorbent materials. In this regard, novel electrospun composite fibers, based on poly β-hydroxybutyrate (PHB) and in situ synthesized poly(vinyl formate) (PVF), loaded with protonated montmorillonite (MMT-H) were prepared via electrospinning in the presence of formic acid, a volatile solvent for polymers and a protonating agent for the pristine MMT-Na. The morphology and structure of electrospun composite fibers were investigated through SEM, TEM, AFM, FT-IR, and XRD analyses. The contact angle (CA) measurements showed increased hydrophilicity of the composite fibers incorporated with MMT-H. The electrospun fibrous mats were evaluated as membranes for removing cationic (methylene blue) and anionic (Congo red) dyes. PHB/MMT 20% and PVF/MMT 30% showed significant performance in dye removal compared with the other matrices. PHB/MMT 20% was the best electrospun mat for adsorbing Congo red. The PVF/MMT 30% fibrous membrane exhibited the optimum activity for the adsorption of methylene blue and Congo red dyes.

## 1. Introduction

During the past decade, the study of biodegradable and bio-based polyesters has gained great popularity, not exclusively for research purposes but also for industrial applications. A distinguished member of the polyhydroxyalkanoate polyester family is poly β-hydroxybutyrate (PHB). It is obtained through multi-step extraction/purification from different bacterial strains or through synthetic pathways [1,2,3,4]. It is favored for being eco-friendly, biodegradable, and compatible with other thermoplastics. Well-known PHB disadvantages include its hydrophobicity, its high degree of crystallinity and inherent brittleness, and its low surface functionality. In this regard, the emerging and already industrially applicable electrospinning technique may offer a convenient solution for acquiring PHB with increased hydrophilicity and functionality upon blending it with polar groups containing polymers, e.g., poly(ethylene oxide) [5] or poly(vinyl alcohol), PVA [6]. The latter is especially desirable, as commercial PVA co-polymers are cheap, industrially available, and rich in functional polar -OH and acetate groups, allowing for further chemical modifications or the compatibilization of different metal oxides and clay nanoparticles [7]. In this regard, PHB/PVA-blend fibrous mats were obtained with the use of volatile perfluorinated alcohol—hexafluoro-2-propanol as a common polymer blend solvent, producing electrospun mats with reduced crystallinity and improving their polarity and hydrophilicity [8]. The polar nature of PVA requires the use of expensive and environmentally/health hazardous PHB compatibilization solvents instead of the traditionally used, cheap, and less environmentally hazardous chlorinated hydrocarbons, e.g., chloroform and dichloromethane, which unfortunately act as anti-solvents for PVA. In this regard, the recent work of Penchev et al. showed the use of 99% formic acid (FA) as an innovative green chemistry solvent for electrospinning pure PHB and its blends with a widely available plant-extracted polypeptide: soy protein. This work also represents a great potential for other polar–nonpolar polymer–inorganic nanoparticle hybrid blends and their compatibilization and subsequent electrospinning [9]. Another known, but rarely explored, feature of FA is its ability to induce the esterification of dissolved PVA in situ under mild conditions with a produced water-insoluble poly(vinyl formate) (PVF) co-polymer [10,11].

Among natural clay materials, sodium montmorillonite (MMT-Na) is a well-known and widely explored material that is used as an effective layered aluminosilicate sorbent for aqueous organic dye removal and water remediation; it is also a polymer reinforcing filler [12,13,14]. This clay exhibits a high specific surface area that interacts with polymeric chains to boost their properties in resulting nanocomposites. Montmorillonite (MMT) has attracted considerable attention as a mineral clay in the field of flake-based hybrids. This is on account of its peculiar features, such as swelling with a high lamellar expansion capacity in aqueous solutions [15]. Montmorillonite is one of the most widely used layered aluminum silicate clays with exchangeable cations and reactive –OH groups on its surface [16]. It is formed in layers, maintaining a platelet structure. The interlayer spacing is nearly 1 nm [17]. Pristine montmorillonite exhibits a hydrophilic nature. The polar nature of MMT-Na is the reason for its incompatibility with non-polar hydrophobic polymers and polyesters, in particular, those without surface hydrophobization treatment and the resultant organo-modified montmorillonites (OMMT), which are intrinsically compatible with polyester matrices. However, the incorporation of OMMT into hydrophobic polymer matrices is a particular and a commonly applied approach in polyester nanocomposite preparation. It does not solve the problem of imparting the needed hydrophilic-hydrophobic balance and increased water sorption capacity desired in manufacturing disposable substrate composite materials for aqueous dye removal applications. Nanoclay-polymer composites exist in either intercalated or exfoliated forms. Some polymeric chains disperse into the nanofiller layers with a steady interlayer spacing, forming intercalated composites [18].

Organic dyes are widely used in the coating industry, the textile industry, the photocatalytic industry, and photochemical applications. Treatment of contaminated and colored wastewater from textile or other industries is an important problem that has attracted researchers’ attention [19]. Different pathways have been followed to remove dyes from aqueous media. They include ion-exchange, membrane separation, and adsorption. Adsorption is considered a facile method because it is easily implemented and produces nontoxic byproducts. As mentioned, aluminosilicate clays such as kaolinite and montmorillonite are frequently used for dye removal applications. However, collecting and abstracting the accumulated dye sorbent in the form of fine dispersion is still a challenge for current technology [20,21,22]. Methylene blue is a typical cationic dye. Various studies have investigated how to remove it from water through adsorption or photochemical decomposition [23,24]. Researchers found that montmorillonite clay is effective as an ion-exchange type adsorbent for the removal of both cationic methylene blue and anionic Congo red dyes [25,26]. Yusef Khaniabadi et al. investigated natural montmorillonite as a low-cost and effective absorbent for Congo red sorption from aqueous solutions and showed that the data were well fitted by pseudo-second order kinetic and Freundlich isotherm models [27]. Another report showed that the removal of acid dyes was improved using sulfuric acid-activated bentonite clay compared with untreated bentonite. They showed that the protonated clays displayed higher adsorption capacities than the natural bentonite clay [28].

Based on our experience and accumulated knowledge, we have designed the present study with the aim of preparing spinnable highly protonated montmorillonite composites based on PHB or PVF, main-chain and side-chain polyesters, respectively. These composites are produced via solution dispersion electrospinning. This new approach could be a promising green chemistry pathway for the use of formic acid as common polymer solvent instead of perfluorinated hydrocarbon solvent alternatives, and its simultaneous use as an efficient dispersant of MMT-H as well. We have found that during the dispersion process, formic acid reacts instantly with natural MMT-Na forms, transforming them into protonated MMT-H forms possessing strong dye sorption properties that act as reactive solvents for the in situ esterification of PVA into PVF co-polymers. The result was preparation of well-dispersed electrospun composite fibrous membranes based on PVF/MMT-H and/or PHB/MMT-H with cationic (methylene blue) and anionic (Congo red) dyes sorption properties. These properties were studied in detail through kinetics and isotherm investigations.

## 2. Materials and Methods

### 2.1. Materials Characterization

Poly β-hydroxybutyrate powder from a bacterial origin with an average molecular mass of 230,000 g·mol^−1^ was kindly supplied by Dr. Urs Hanggi (Biomer, Krailling, Germany). Poly(vinyl alcohol) fine powder with a DH of 97.5–99.5% and a Mw avr. = 72 000 g·mol^−1^ from Merck-Schuchardt (Hohenbrunn, Germany)) was used. Pure formic acid (Merck, 99%) was used as a solvent. Natural montmorillonite clay (MMT-Na) was supplied by Southern Clay Products Inc, USA. For electrospinning experiments, a commercial Genvolt HV PSU 30 W direct current power supply of 0–30 kV and an Alaris IVAC^®^ P3000 medical syringe pump were used. Wide-angle X-ray diffraction (WAXD) scans were obtained with a Bruker D8 Advance ECO diffractometer in reflection mode over a 2θ range of 5–50°. The ATR FT-IR analyses were performed on a Thermo Scientific Nicolet iS50 apparatus in the range of 400–4000 cm^−1^. Thermogravimetric analyses were performed in an argon atmosphere on a TGA-4000 Perkin Elmer instrument (temperature range 30 °C–800 °C, 10 °C/min, nitrogen atmosphere, 30 mL·cm^−1^). SEM analysis was performed on a high-resolution scanning electron microscope (SEM) (QUANTA FEG 250 ESEM). TEM analyses were carried out on an HR STEM JEOL JEM 2100. AFM imaging was performed on a Bruker Dimension Icon microscope working under peak force tapping mode. The statistical measurements of fibers were performed with Image J software. The contact angle values were recorded with a camera attached to a computer. Deionized water droplets (5 µL) were transferred to the surface of the membrane at 25 °C to explore the membrane hydrophilicity.

### 2.2. Preparation of PVF- and PHB/MMT-H Spinning Dispersions

In order to prepare electrospinable MMT dispersion solutions, the first step includes preparing a stock-spinning solution of in situ-formed PVF. This was performed by dissolving 0.5 g of PVA powder in 9.5 g FA at 60 °C for 3 h under constant stirring. The obtained viscous solution was left to stay at room temperature (25 °C) for 24 h before electrospinning experiments in order to reach a high degree of esterification. The degree of esterification (DE) for the obtained PVF co-polymer was determined gravimetrically by casting a known amount of the resultant PVF solution with a doctor’s blade (0.1 mm gap) onto a glass substrate. Sol-gel immersion of the film in a water bath and subsequent vacuum oven drying were carried out for the inversed gel film at 70 °C overnight. The experimentally determined DE of the pristine PVA was 80%. The spinning dispersion of PVF with 30 wt.% MMT content was performed by dispersing 0.25 g MMT-Na in 9.5 g FA using a laboratory vortex followed by an additional 10 min ultrasound bath treatment. The obtained pristine in situ-formed protonated montmorillonite clay (MMT-H) was further sterically stabilized by dissolving 0.5 g PVA according to the described procedure. The final milky white fine dispersion was left overnight under constant stirring before electrospinning. Stock dispersions of MMT-H (10 and 20 wt.% on polymer weight base) in PHB were prepared by adding and sonicating 0.05 g (for PHB/MMT 10% composite) or 0.1 g (for PHB/MMT 20%) of pristine MMT-Na in 7 g FA. This step was followed by the addition of 0.5 g PHB powder after 5 min of ultrasound treatment until a homogeneous dispersion of both components took place.

Thereafter, the common particle dispersion of MMT/PHB was heated in a silicon oil bath at 80 °C for 10 min under magnetic stirring until the PHB particles were fully dissolved. The resulting MMT dispersion in PHB was left to cool down to 40 °C before electrospinning. The control pristine PHB electrospinning solution was prepared in the same manner without the addition of MMT-Na. The PHB/PVF/MMT 10% (component weight ratio: 63:27:10%) spinning solution was prepared by mixing and homogenization 3 g of PVF/MMT 30 stock dispersion with 10 g of 5 wt.% solution of PHB in FA at 60 °C. After obtaining a homogeneous mixture, the spinning solution was left to cool down to 40 °C before the electrospinning experiment.

### 2.3. Electrospinning Process

The electrospinning experiments were carried out with a fixed applied voltage of 20 kV and a stationary aluminum collector (20 × 20 cm, 2 mm thickness). The spinning solution flow rates and the distance between the electrodes were varied, depending on the electrospun sample polymer/MMT composition, as follows: PVF-0: 0.2 mL·h^−1^, 15 cm; PVF/MMT 30%—0.5 mL·h^−1^, 15 cm; PHB/MMT 10%—0.7 mL·h^−1^, 20 cm; PHB/MMT 20%—0.5 mL·h^−1^, 20 cm; and PHB/PVF/MMT 10%—1.5 mL·h^−1^, 15 cm. The composite mats were immersed for 24 h in water to remove the residual sodium formate salt then dried in a vacuum chamber. In Table 1, the compositions of different samples and their electrospinning parameters are summarized.

### 2.4. Dye Sorption and Kinetics Studies of the Composite Membranes

The adsorption experiments of cationic dye (methylene blue) and anionic dye (Congo red) for the investigated membranes were conducted at room temperature (25 ± 2 °C) under different contact time intervals (0–240 min). The initial dye concentration ranged from 10–50 mg/L. The adsorption experiment was designed as a batch in stoppered reagent bottles at a constant dose of 1 g/L. The shaking speed was 150 rpm. The concentrations of methylene blue and Congo red before and after adsorption were determined using a UV-Visible Spectrophotometer (Cary 100 UV-Vis) at wavelengths of 665 and 498 nm, respectively.

The adsorption capacity (*qe* in mg/g) was calculated by the following equation:

(1) $$qe=\frac{Co-Ce}{m}*V$$

where *Co* and *Ce* are the initial and final dye concentrations (mg/L), *V* is volume of dye solution (L), and m is the weight of the adsorbent fiber mats (g). This study examined the interactions between pollutants (dyes) and the nanofiber membranes using adsorption isotherm models. The Freundlich and Langmuir models are the two adsorption isotherm models which are commonly used. In order to identify the best model for our purposes, linear regressions of Langmuir (Equation (2)) and Freundlich (Equation (3)) models were used.

(2) $$C_{e}/q_{e}=C_{e}/q_{max}+1/(q_{max}K_{L})$$

(3) $$\ln q_{e}=\frac{1}{n}\ln C_{e}+\ln K_{F}$$

where *C_e_* is the equilibrium concentration of the dye in the solution (mg/L) and *q_e_* stands for the adsorption capacity at equilibrium (mg/g). *q_e_* was calculated from triplicate measurements for three separate replicates of each experiment. *q_max_* is the maximum adsorption capacity (mg/g), *K_L_* and *K_F_* are the adsorption rate constants of the Langmuir and Freundlich models, respectively, and n is the heterogeneity parameter [29]. Using linear regression, correlation coefficients (R^2^) were estimated and the adsorption isotherm models were evaluated for applicability.

#### Adsorption Kinetics

Two kinetic models, pseudo-first order and pseudo-second order equations, were used to assess the rate of dye adsorption in the prepared PVA nanocomposites mats [30,31,32]. The pseudo-first order equation was represented as follows:

(4) Ln (qe−qt)=Ln qe−k1 t

where *k*1 (1/min) is the rate constant per minute and *qe* and *qt* (mg/g) are the amount of adsorbed dye at equilibrium and at time *t* (min), respectively.

The pseudo-second order equation was represented as follows:

(5) $$\frac{t}{qt}=\frac{t}{k2qe^{2}}+\frac{t}{qe}$$

where *k*2 (g/mg.min) is the pseudo-second order rate constant and *qe* (mg/g) is the quantity of adsorbed dye at equilibrium.

## 3. Results and Discussion

### 3.1. Characterization of the Electrospun Membranes

**Figure 1:**
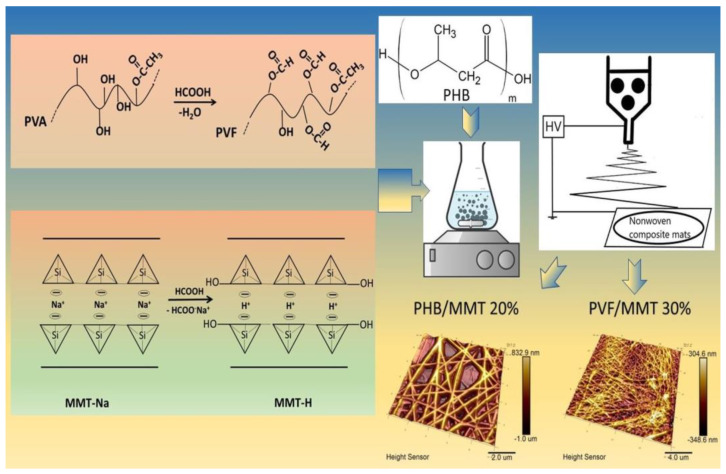
General materials preparation scheme of the novel dye sorption electrospun membranes.

In the present study, we explored the ability of the volatile and highly polar 99% formic acid to disperse natural montmorillonite (MMT-Na) effectively and convert it to protonated MMT-H in situ. Hence, an ion-exchange reaction takes place with the formation of sodium formate salt as a byproduct. Moreover, FA acts as a highly efficient reactive solvent for the in situ esterification of water-soluble pristine PVA co-polymers. It also tends to be water insoluble as an amphiphilic poly(vinyl formate) co-polyester. These two general properties led us to the idea to use FA as a green chemistry alternative to the state of the art perfluorinated low-alcohols and the trifluoracetic acid solvents for the electrospinning of PVF/MMT 10%, PHB/MMT 20%, PHB/MMT 30%, and the PHB/PVF/MMT (70:30/10 wt.%) composites, as illustrated through the general materials preparation scheme in Figure 1. The latest composite will be further referred to as PHB/PVF/MMT 10%.

Compatibilization of polar MMT-Na with the use of traditional aprotic solvents (e.g., chloroform and dichloromethane) for biodegradable polyester (e.g., PHB) dissolution is a challenging task. The most commonly used approach in this case is known from the literature on polyester compatibilization and involves blending with hydrophobic organo-modified montmorillonites [33]. However, with this common approach, the resultant composites retain, or even increase, their hydrophobicity. Thus, they could be considered mostly unsuitable as aqueous media dyes and heavy metal ion removal sorbents. In this regard, the works of Iordanskii et al. and Penchev et al. exploring the use of pure formic acid or its mixture with chloroform as green chemistry polar (co-)solvents demonstrated their applicability for electrospinning of pure PHB or PHB blends with soy protein [9,34]. These pioneering works were the base for further research into the possible compatibilization of natural MMT-Na, which has been already mentioned, and effective aqueous dye removal sorbents. This feature is due to MMT-Na’s layered platelet structure and ion-exchange properties [12]. The immobilization of MMT in high specific surface electrospun biodegradable fibrous matrices is beneficial for the safe removal of sorbed organic dye contaminants from the environment and for the utilization of nanoclay composite membranes in disposable biodegradable forms. In this regard, our experiments started with the use of pure formic acid (FA) solvent, which acts as both a protonating agent for converting sodium MMT to protonated MMT-H and as a dispersion media. Simultaneously, we used FA as a reactive solvent for the in situ esterification of pristine water-soluble PVA to water-insoluble side-chain polyester PVF, which disperses commonly with MMT-H. Thereafter, FA sterically stabilizes the dispersed clay particles. We found that the obtained PVF solution can effectively stabilize up to 30 wt.% MMT-H clay. The resulting highly concentrated stock solution can be used for the preparation of highly loaded polymers with MMT-H composite fibers (PVF/MMT 30%). Moreover, it may be mixed with PHB solution to obtain polymer composite fibers with lower MMT-H contents (PHB/PVF/MMT 10%) via electrospinning. Furthermore, two different pure PHB composite fibers with 10% and 20 wt.% MMT-H were incorporated in the preparation from formic acid solution. The lower solution viscosity of PHB and its main-chain polyester nature can lead to a maximum loading of MMT-H in which stable electrospinning occurs upon loading 20 wt.% clay.

**Figure 2:**
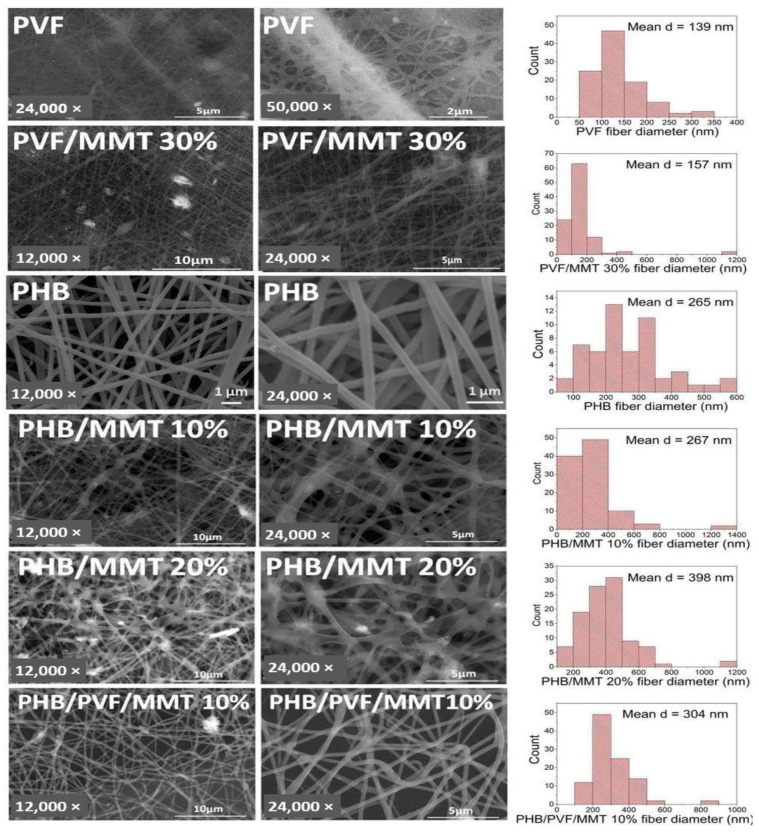
SEM images of PVF, PVF/MMT 30%, neat PHB, PHB/MMT 10%, PHB/MMT 20%, and PHB/PVF/MMT 10%, with the average diameter distribution of these fibers.

In Figure 2, SEM micrographs of the electrospun mats are presented. The average fiber diameters determined via SEM are as follows: 139 nm ± 53 (PVF); 157 nm ± 119 (PVF/MMT 30%); 265 nm ± 105 (PHB); 267 nm ± 161 (PHB/MMT 10%); and 304 nm ± 109 (PHB/PVF/MMT 10%). As can be seen, the morphology of the composite fibers is affected by the incorporation of proton-conducting MMT-H. There is an observed increase in the average fiber’s diameter with the formation of more defects and branched structures. However, adding the second side-chain polyester component, PVF, led to a significant increase in the smoothness and homogeneity of the PHB/PVF/MMT 10% fibers compared with the PHB/MMT 10% and PHB/MMT 20% composite fibers, which, as mentioned, showed some bead defects and partially intercrossing fibrous structures. This could be explained by the more flexible chain nature of PVF co-polymers and some additional intermolecular wrapping between PHB and PVF macromolecules in FA solution.

**Figure 3:**
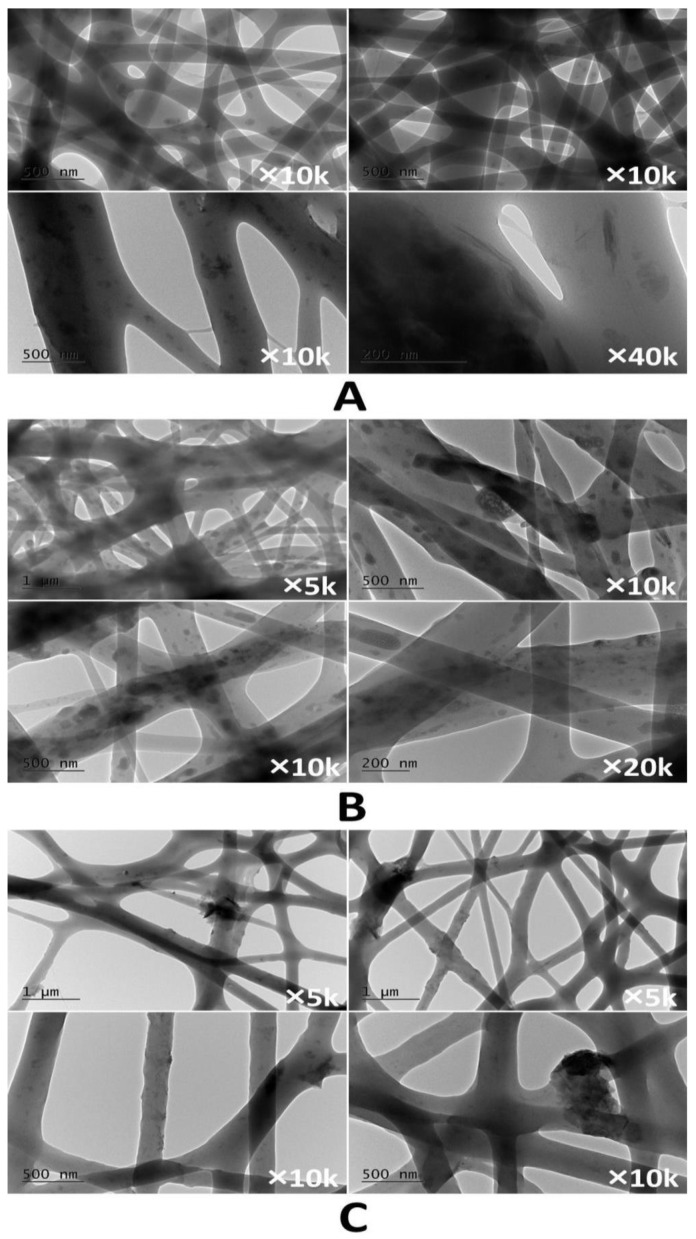
TEM images of (**A**) PHB/MMT 10%; (**B**) PHB/PVF/MMT 10%; (**C**) PHB/PVF/MMT 10% after aq. MB sorption.

The TEM analyses of the investigated membranes are depicted in Figure 3. The composite PHB/MMT 10% fibers in Figure 3A show that mostly exfoliated MMT nanosheets are incorporated into the fibrous polyester matrix. In the case of composite PHB/PVF/MMT fibers, some nanophase clusters are distributed along the fibers’ axes (Figure 3B). Figure 3C shows that the TEM micrographs of the PHB/PVF/MMT mat after 20 min in contact with aqueous 1 mg/L methylene blue solution revealed a swelling in the composite fibers. Moreover, the presence of expanded hydrated MMT clusters along the fiber axis is proof of their good sorption property.

**Figure 4:**
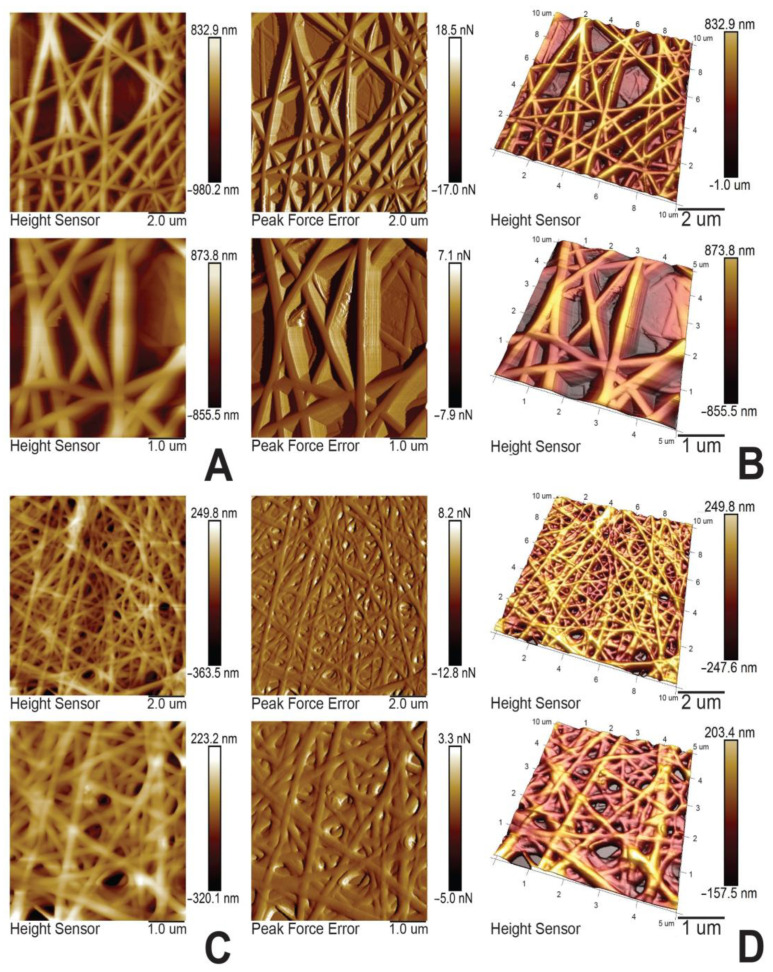
(**A**) AFM 2-D images of PHB/MMT 20%; (**B**) AFM 3-D images of PHB/MMT 20%; (**C**) AFM 2-D images of PVF/MMT 30%; and (**D**) AFM 3-D images of PVF/MMT 30%.

**Figure 5:**
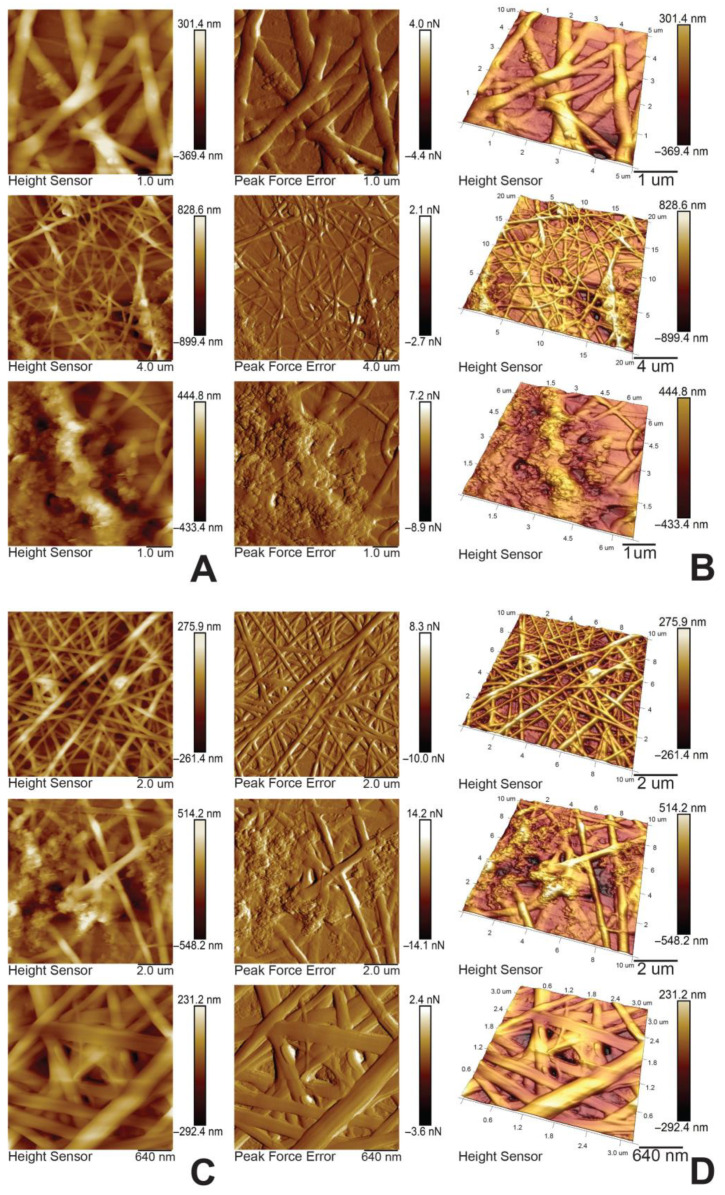
(**A**) AFM 2-D images of PHB/MMT 20%; (**B**) AFM 3-D images of PHB/MMT 20%; (**C**) AFM 2-D images of PVF/MMT 30%; and (**D**) AFM 3-D images of PVF/MMT 30% after MB sorption.

The 2-D and 3-D AFM surface topography images of the PHB/MMT 20% and PVF/MMT 30% composite electrospun fibrous mats are shown in Figure 4A–D. As can be seen, the PHB/MMT 20% mat forms a loose, smooth-surfaced fibrous membrane structure (Figure 4A,B) compared with the very dense, small-diameter, well-overlapped fibrous structure of PVF/MMT 30% composite membranes (Figure 4C,D). The AFM surface topography images of the MMT-H-containing composite fibrous membranes after 10 h of contact with aqueous 1 mg/L methylene blue solution showed significant changes in both samples (Figure 5A–D). A noticeable swelling of the fibers is observed after contact with the aqueous dye solution, which is in accordance with the TEM observation. The 2D and 3D surface morphology of the PHB/MMT 20% mat after water contact revealed swelling of the polymer matrix and less sharp fiber contrast morphology compared with the dry pristine mat (Figure 4 and Figure 5A,B). In both dye-adsorbed composite polyester fiber samples, some random sections of expanded MMT particles over the fiber surface are visible. This could be confirmation of the already observed results of the TEM analysis of MMT platelet clustering.

**Figure 6:**
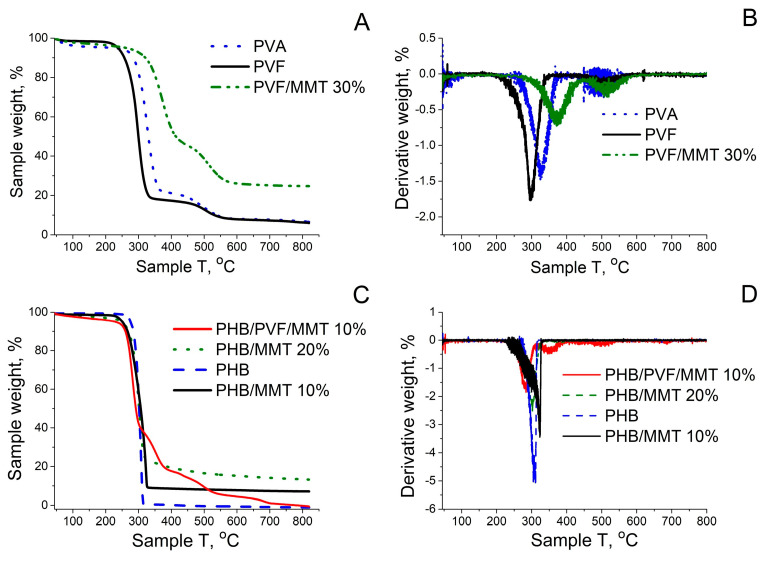
(**A**) TG and (**B**) DTG curves of PVA, PVF, and PVF/MMT 30%; (**C**) TG and (**D**) DTG curves of PHB, PHB/MMT 10%, PHB/MMT 20%, and PHB/PVF/MMT 10%.

The TGA and DTG curves of the starting materials and the composite electrospun polyester fibers with incorporated MMT-H are clarified in Figure 6. Three stages are registered during the thermal degradation of both pristine PVA and the in situ esterified mats by the formic acid solvent PVF co-polymer. The lower mass loss (1.81%) of PVF below 200 °C in comparison with that of pristine PVA is due to an elevated hydrophobicity. It results from the FA esterification of the hydrophilic -OH groups of PVA. The first weight loss (4.17%) of PVA below 200 °C was attributed to the evaporation of free water molecules in PVA due to its hydrophilic property [35]. The second step of decomposition is attributed to main chain degradation and the generation of CO, CO_2_, and volatile hydrocarbons from the PVF matrix. The third step of degradation of PVF is assigned to the carbonization process, with a small fraction of char formation. The increased thermal stability of PVF/MMT 30% and the slightly increased stability of the PHB/MMT 10% electrospun composite membranes compared to pristine PVF and PHB fibers may be due to the incorporation of the well-dispersed MMT-H clay in the polymer matrix of electrospun mats. This effect is most profound in the PVF/MMT 30% sample. MMT improves the structural stability of polymer fibers [36]. The resulting residue above the degradation temperature of the polymers is associated with the presence of thermally intact montmorillonite and a small fraction of charred material.

**Figure 7:**
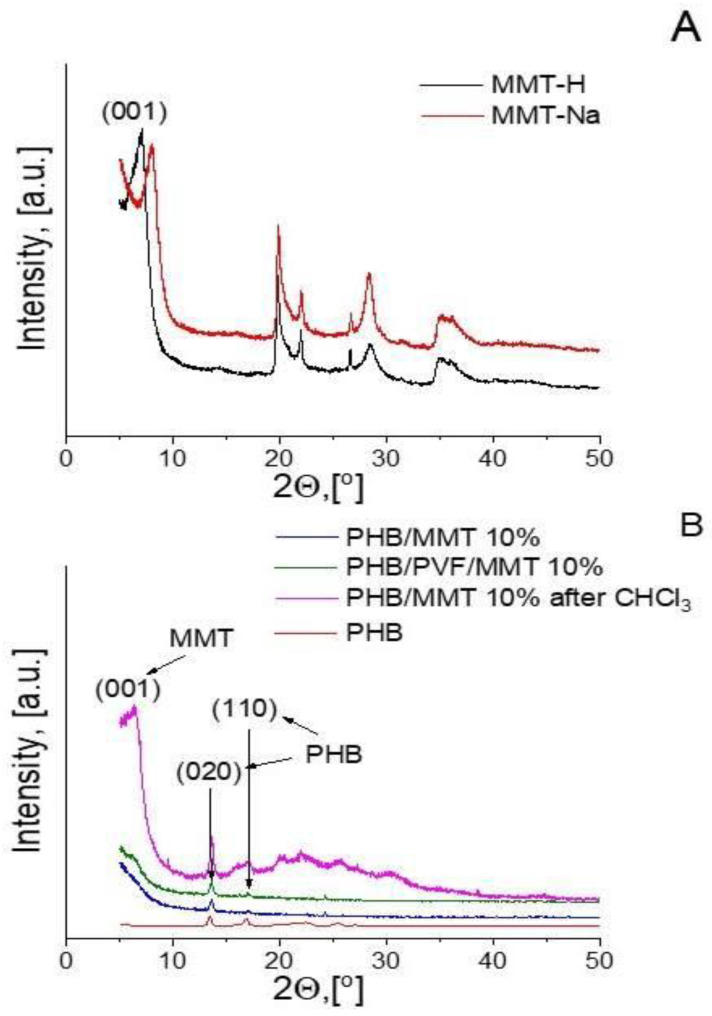
XRD patterns of natural/protonated montmorillonite (**A**) and electrospun composite fibers with 10 wt.% incorporated MMT-H before and the sample PHB/MMT 10% was extracted with chloroform (**B**).

The XRD analyses of pristine MMT-Na and the in situ protonated MMT-H form obtained after the ion-exchange reaction with formic acid during the dispersion process are presented in Figure 7A. The structure of the nanocomposites is demonstrated by the enlargement degree of d001-spacing. The protonation of MMT-Na in the presence of formic acid shifts the d001 to lower angles, from 2θ = 7.88° (d001 = 11.21 nm) in MMT-Na to 2θ = 7.02° (d00 = 12.58 nm). The XRD patterns of electrospun composite fibers, namely PHB/MMT 10% and PHB/PVF/MMT 10%, show a broad amorphous halo with two main diffraction peaks at 13.4° and 16.8°. They correspond to the orthorhombic crystal structure of PHB (Figure 7B). The XRD patterns of PHB/PVF/MMT 10% and PHB/MMT 10% indicate an exfoliation of MMT-H platelets for which there is a lack of a d001 diffraction peak. In order to prove further the existence of exfoliated MMT polymer composites obtained during the electrospinning process, we performed an experiment in which we dissolved a free-standing fibrous mat sample of PHB/MMT 10% in polar aprotic solvent (chloroform). After contact between the mat and the chloroform, almost immediate dissolution of the PHB matrix (2 wt. %) takes place. The obtained solution has a milky opalescence, which is an indication of the colloidal dispersion of the MMT-H in the PHB/chloroform solution [37]. After that, the chloroform dispersion was poured into a Petri dish and the solvent was evaporated, resulting in a free-standing thin composite film. The XRD analysis of this sample revealed the original d001-spacing of MMT-H. This is an indication, to some degree, of the secondary aggregation of the re-dispersed polar MMT-H platelets from the original fiber exfoliated MMT form. This property is due to the less polar nature of chloroform solvent compared to the protic formic acid solvent. A prominent re-crystallization and increased crystallinity degree of the PHB film matrix after chloroform evaporation was also observed. This is in contrast with the low-crystallinity electrospun PHB-based composite fibers obtained through the fast evaporation of formic acid dispersions explored in this study during the electrospinning process.

**Figure 8:**
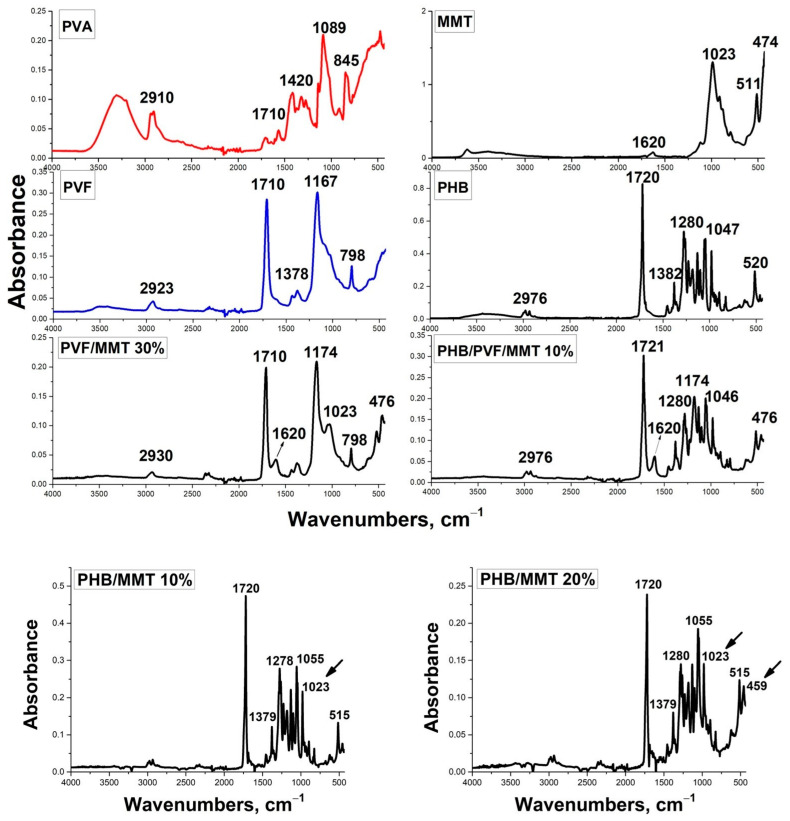
ATR FT-IR analysis of the starting materials and the composite electrospun fibers.

In Figure 8, the ATR FT-IR spectra of the prepared electrospun composite membranes are revealed. The band at 1720–1721 cm^−1^ is attributed to the C=O stretch of the ester group. The peak at 1278–1280 cm^−1^ is assigned to the ester bonding observed in the FT-IR spectra of PHB, PHB/MMT 10%, PHB/MMT 20%, and PHB/PVF/MMT 10% [38]. The registered peaks at 2910 cm^−1^ and 1420 cm^−1^ are due to the vibration of C–H from alkyl groups and the bending vibration of CH_2_ groups in PVA. The vibration band at 1710 cm^−1^ refers to the stretching C=O vibration from unhydrolyzed acetate groups in the pristine PVA co-polymer. The band at 1089 cm^−1^, which corresponds to the stretching vibrations of C-O-H in pristine PVA, almost disappeared after in situ esterification with formic acid. The stretching vibration of the carbonyl group associated with the sharp and intensive peak around 1710 cm^−1^ is characteristic for PVF. It shows the high degree of in situ esterification of the PVA precursor. The CH group deformation vibrations of the formyl group are registered at 1380 cm^−1^. The peak observed at 798 cm^−l^ is attributed to rooking CH_2_ vibrations. The band observed at 1167–1174 cm^−1^ is associated with the C-O stretching vibration in PVF, PVF/MMT 30%, and PHB/PVF/MMT 10% [38]. The absorption bands in the range of 515–474 cm^−1^ are due to the deformation vibrations of Si–O-Al and Si–O–Si. In FT-IR spectra of PVF/MMT 30% and PHB/PVF/MMT 10%, an absorption band is observed at 1620 cm^−1^, associated with the bending vibrations of the -OH group of water molecules present in the montmorillonite [39,40,41].

**Figure 9:**
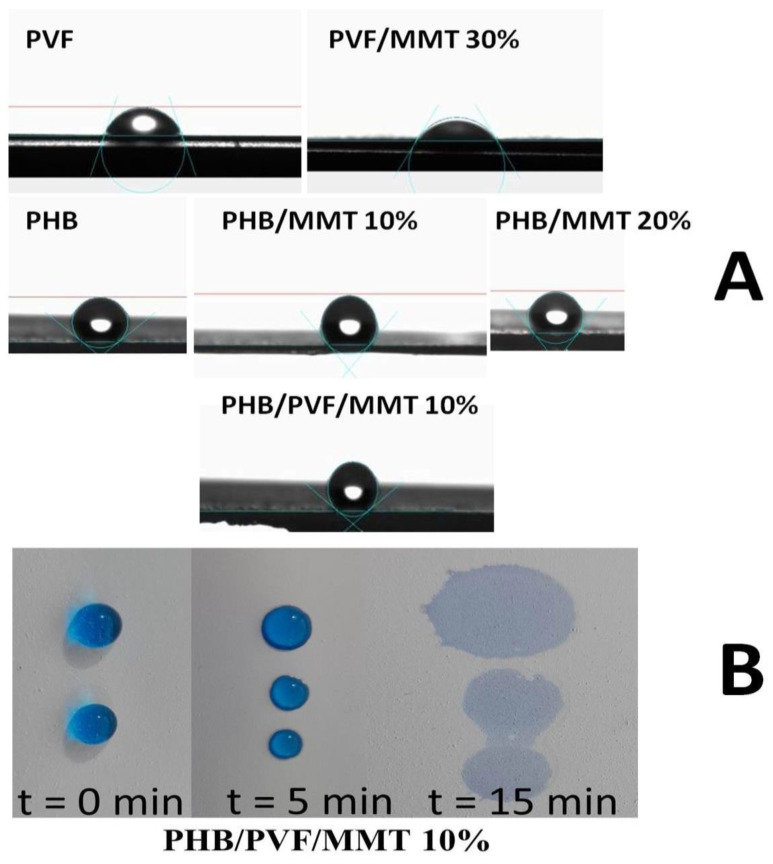
(**A**) Contact angle(s) of PVF, PVF/MMT 30%, neat PHB, PHB/MMT 10%, PHB/MMT 20%, and PHB/PVF/MMT 10%. (**B**) Digital pictures of PHB/PVF/MMT 10% composite membrane in contact with aqueous MB solution at different time intervals.

The measured contact angles of the investigated membranes are shown in Figure 9A. Commonly, PVA exhibits a noticeable hydrophilicity. However, after it undergoes an esterification reaction with formic acid, the hydroxyl groups in PVA change into ester groups. Hence, the formed poly(vinyl formate) (PVF) partially loses its hydrophilic behavior, tending to become a hydrophobic matrix. It displays a contact angle of 70.7°. Upon loading PVF with MMT, we can observe a decrease in the contact angle to 57.5°. This lower value signifies a wetting character for this membrane due to the incorporation of hydrophilic MMT. The potent electrostatic impact of the negatively charged surface of MMT allows it to adsorb positively charged surfaces such as cationic dyes. Neat PHB showed a contact angle of 138°. Upon investigating the PHB/MMT 10% sample, it showed a contact angle of 131.4 °C. This value indicates the dominance of a hydrophobic surface owing to PHB in this membrane, even in the presence of MMT. The PHB/PVF/MMT 10% composite had a higher contact angle value of 142.2° when compared to that of PHB/MMT 10%. At higher MMT content, PHB/MMT 20% displayed a CA value of 125°. Figure 9B displays digital images tracking the spread of MB aqueous solution on the surface of the PHB/PVF/MMT 10% composite at different time intervals from t = 0–15 min, showing an adequate wetting behavior due to the presence of MMT. The contact angle value comparison is between the blended PHB/PVF/MMT 10% and PHB/MMT 10% composite mats. In both cases, the predominant hydrophobization role is played by the more hydrophobic PHB homopolymer, rather than the more polar (amphiphilic) PVF ter-polymer, which contains the more polar formate ester, some free -OH and acetate groups. Thus, the observed slight increase in the contact angle/hydrophobicity of the blended PHB/PVF/MMT fiber mats compared to single polymer PHB/MMT 10% could be attributed mostly to the blend fibers’ smoother morphology and higher fiber diameter compared with those of PHB/MMT fibers.

### 3.2. Adsorption Isotherms and Kinetics Investigations

**Figure 10:**
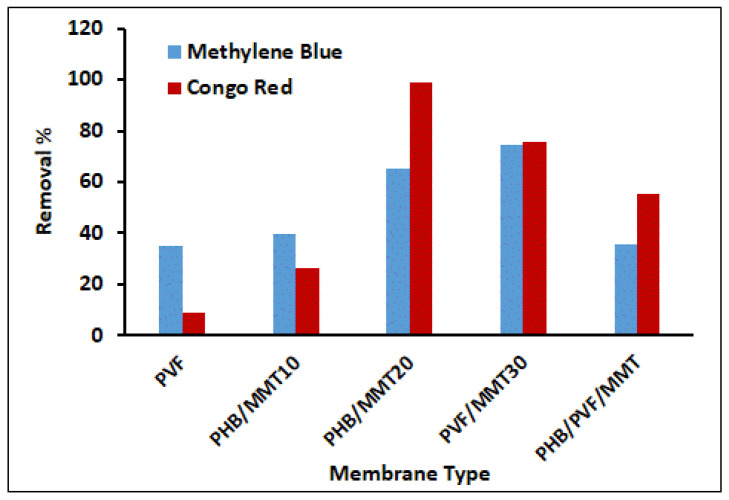
A comparison between the adsorption efficiencies of different electrospun membranes at 25 °C over a period of 24 h with 1 mg of adsorbent and 10 mL of Congo red and methylene blue (10 ppm).

All nanofiber mats were evaluated as membranes for removing cationic and anionic dyes (Figure 10). PHB/MMT 20% and PVF/MMT 30% showed significant performance in dye removal compared with the other matrices. PHB/MMT 20% was the best electrospun mat for adsorbing Congo red. Meanwhile, PVF/MMT 30% exhibited the optimum activity for the adsorption of methylene blue and Congo red. This property may be attributed to the presence of two different charges on the mat surface. Moreover, it may be attributed to the high surface area and the highest MMT-H content afforded by the PVF/MMT 30% composite mat, which possesses a smaller nanofiber diameter (average diameter of 157 nm).

**Figure 11:**
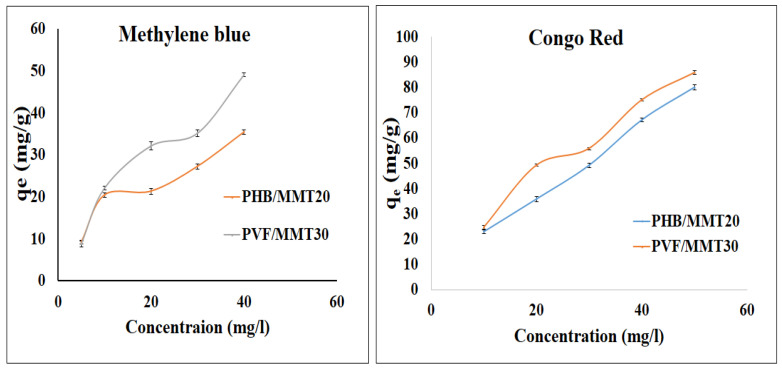
Effect of the concentration of methylene blue and Congo red dyes on adsorption in 1 mg samples of the selected fiber mats, using 10 mL of dye solution at 25 °C over 24 h.

Figure 11 indicates the effect of initial dye concentration on the adsorption capacity of the selected nanofiber membranes. The adsorption capacity of methylene blue increased with the elevating the dye concentration, with an enhanced performance for the nanofibers with 30% clay content. Meanwhile, the adsorption capacity boosted upon increasing the Congo red concentration. The adsorption of PVF with 30% clay was higher than the adsorption of fibers with 20% clay, indicating that the higher clay content corresponds to higher adsorption capacity. The increase in dye concentration may be the result of greater diffusion of pollutants into the active site within the adsorbent and increased saturation at these sites. In the case of the dye concentrations of 20 and 30 ppm, the adsorption capacity values were almost close to each other. This behavior may be attributed to the saturation of the monolayer active sites. Then, the adsorption capacity increased by increasing the dye concentration, most likely because of the π-π attraction between the dye molecules. The pollutants may form stalks and make multilayers that lead to an increase in adsorption capacity. Figure 11 also shows that the adsorption of Congo red is much higher than that of methylene blue, pointing to the higher affinity of both polyvinyl alcohol and clay towards anionic dyes. This may be due to the electrostatic attraction between the anionic dyes and the protonated groups of the composites.

**Figure 12:**
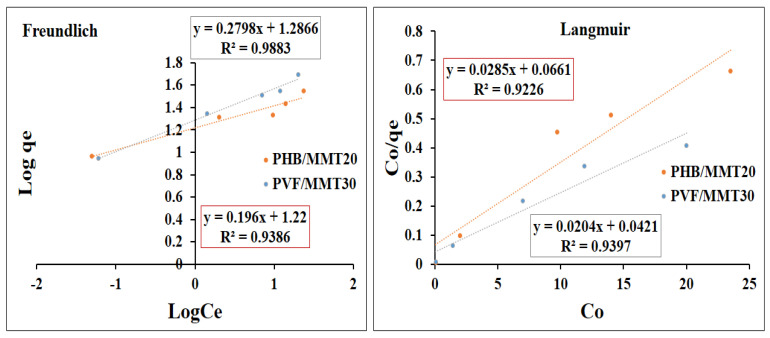
Adsorption Freundlich and Langmuir isotherms for the adsorption of methylene blue on the selected membranes.

**Figure 13:**
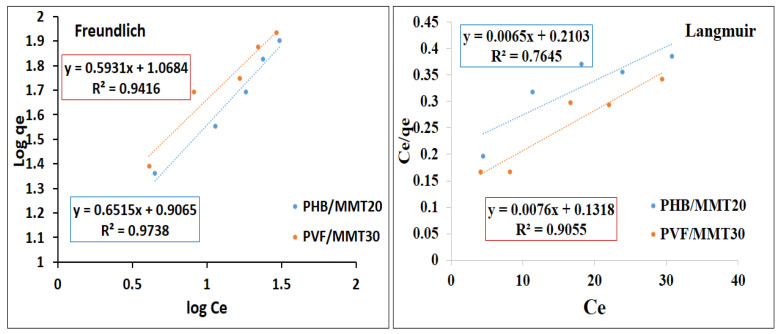
Adsorption Freundlich and Langmuir isotherms for the adsorption of Congo red on the selected membranes.

This study examined the interactions between pollutants (dyes) and nanofiber membranes using adsorption isotherm models. The Freundlich and Langmuir models are the two adsorption isotherm models which are commonly used. In order to identify the best model for our purposes, linear regression of the Freundlich and Langmuir models was used. Correlation coefficients (R^2^) were estimated and the adsorption isotherm models were evaluated for applicability. Figure 12 and Figure 13 show the linear forms of the Freundlich and Langmuir isotherms for methylene blue and Congo red as models of cationic and anionic dyes, respectively. Generally, the Langmuir isotherm model assumes that monolayer adsorption takes place on surfaces with uniformly distributed adsorption sites and without adsorbate transmigration. Accordingly, once the surface approaches a saturation point, it will achieve maximum adsorption [42,43]. In contrast, the Freundlich model represents an adsorption process on heterogeneous surfaces through multilayer adsorption [44]. Methylene blue adsorption on both membranes is illustrated in Figure 12. The adsorption on the PHB/MMT 20% membrane was fitted with Freundlich and Langmuir isotherms according to correlation coefficient values (R^2^) of 0.92 and 0.93, respectively. For PVF/MMT 30%, the Freundlich model has a better fitting (R^2^ = 0.98) than the Langmuir model (R^2^ = 0.93). As shown in Figure 13, Congo red adsorption on PVF/MMT 30% seems to be fitted with both models. However, there was a higher correlation coefficient value (R^2^) for the Freundlich isotherm (0.94) compared to the Langmuir isotherm (0.90). This may suggest that that monolayer adsorption takes place by electrostatic attraction on the active site of PVF/MMT 30%, with multilayers of Congo red stacked over [42]. For PHB/MMT 20%, it is obvious that the adsorption of Congo red is well-fitted to the Freundlich model (R^2^ = 0.97). Here, the hydrogen bonding and the π-π bonding between the Congo red molecules assisted in maximizing the adsorption capacity of the PHB/MMT 20% surface.

**Figure 14:**
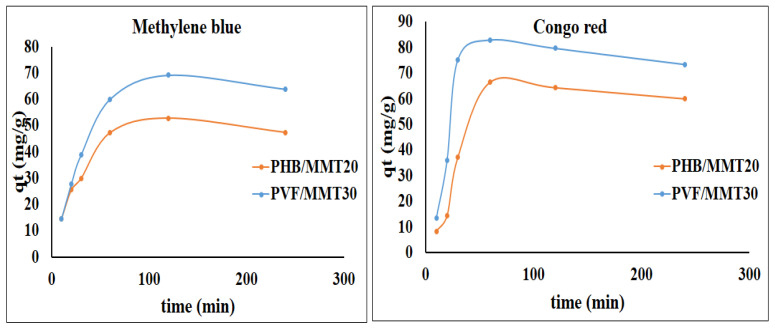
Effect of adsorption time on the adsorption capacity of methylene blue and Congo red using the prepared nanofiber membranes.

Figure 14 displays the effect of contact time on the adsorption capacity of the investigated electrospun membranes towards methylene blue and Congo red dyes. It is clearly seen that the maximum adsorption time was attained after 60 min for both methylene blue and Congo red, after which the adsorption capacity reached a steady state. Furthermore, after contact for more than 90 min, the adsorption decreased. This behavior may be correlated to the saturation of the active site of the dye binder. In other words, the site cannot bind anymore, as the dye molecules tend to desorb out from the fiber mat, bringing out a slight decrease in the adsorption capacity. The adsorption capacity likely increased upon elevating the dye concentration. This behavior can be correlated to the chemi- and physi-sorption of dye molecules forming a multilayer of adsorbate. The removal efficiency decreased as the dye concentration increased, with optimum balances at 40 ppm for Congo red and 30 ppm for MB dyes, respectively. Thereafter, the removal percentage decreased extensively and the adsorption capacity decreased slightly. Therefore, this decline in adsorption may be related to the desorption of the aggregated multi-layer dye molecules. After a long time (4 h), the dye molecules adhering through weak Van Der Waals forces started to detach from the matrix.

**Figure 15:**
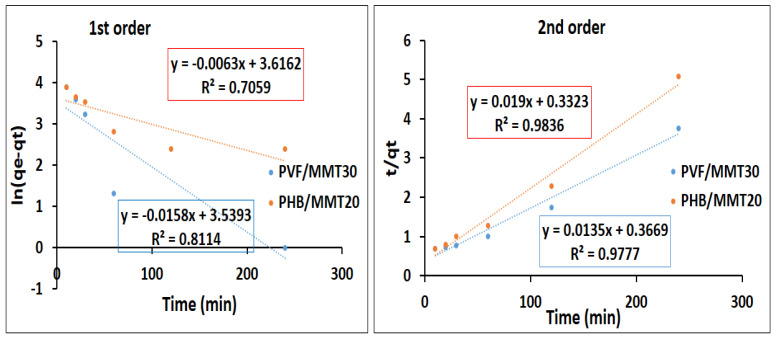
Pseudo-first and second order kinetics for the adsorption of methylene blue using nanocomposite membranes.

**Figure 16:**
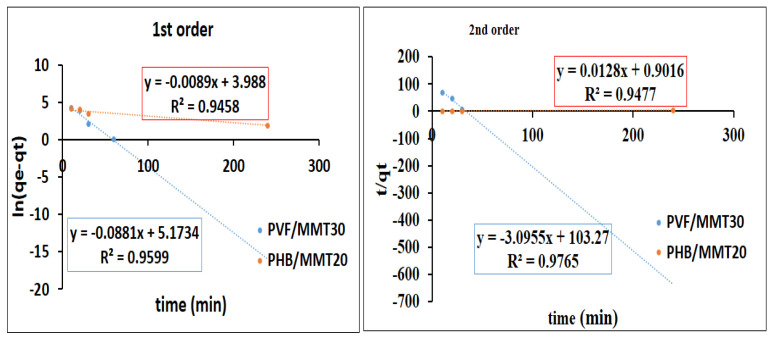
Pseudo-first and second order kinetics for the adsorption of Congo red using nanocomposite membranes.

Pseudo-first order and pseudo-second order kinetic models were employed to evaluate the dye adsorption rate of the tested membranes. The rates of the adsorption for methylene blue and Congo red on the synthesized membranes were assessed by pseudo-first order and second order kinetics models. The results are shown in Figure 15 and Figure 16. As demonstrated in Figure 15, the adsorption of methylene blue on both PVF/MMT 30% and PHB/MMT 20% fitted with the second order kinetic model, as indicated by the R^2^ for both membranes (0.9777 and 0.9836, respectively). The qmax derived from the second order (74 and 52) are closer to the experimental values of 69.1 and 52.7 for the two adsorbents, PVF/MMT 30% and PHB/MMT 20%, respectively. Figure 16 indicates that the rate of adsorption of Congo red was mostly fitted with the second order model for the two membranes, whereas the value of R^2^ was 0.9765 and 0.9477 for PVF/MMT 30% and PHB/MMT 20%, respectively. These results indicate that rate of adsorption depends on both the adsorbent nanofibrous membranes and the organic dyes (the cationic methylene blue and the anionic Congo red).

**Figure 17:**
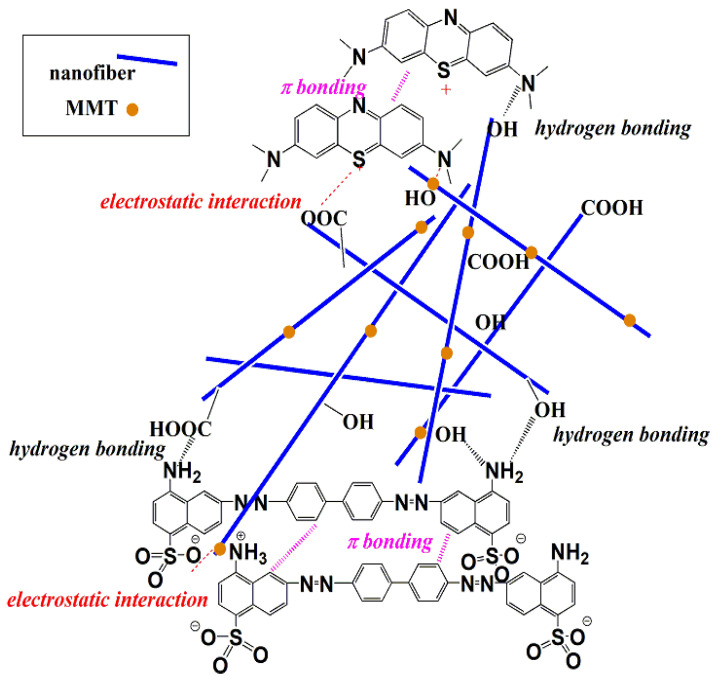
A schematic representation of the proposed adsorption mechanism for the dye on the surface of the nanofiber.

A proposed adsorption mechanism can be suggested which functions through electrostatic attraction between the active groups of the anionic or cationic dyes with the active sites on the nanofiber surface. Then, the dye molecules accumulate on each other via π-π bonding, as illustrated in Figure 17.

## 4. Conclusions

In this study, novel composite mats electrospun from PHB and/or in situ PVF, biodegradable polyesters, and their composites, highly loaded with in situ protonated montmorillonite MMT-H, were prepared and characterized. Formic acid was utilized as a reactive solvent for MMT-Na protonation and as an effective clay particle dispersant. The introduction of MMT-H nanofiller into the fibrous membrane polymer matrix leads to the increased hydrophilicity and wettability of the electrospun fibers in contact with aqueous dye media. The dye sorption isotherms and kinetics studies of the investigated nanocomposite fibrous mats showed good potential as biodegradable and disposable fibrous sorbent membranes for the removal from contaminated water of both cationic and anionic dyes, e.g., methylene blue and Congo red. The adsorption capacity of the prepared membranes for MB and CR dye removal boosted noticeably upon raising the MMT content. The adsorption of methylene blue on both PVF/MMT 30% and PHB/MMT 20% fitted with the second order kinetic model, as indicated by R^2^ for both membranes (0.9777 and 0.9836, respectively). In addition, the rate of adsorption of Congo red was mostly fitted with the second order model for the two membranes. R^2^ showed values of 0.9765 and 0.9477 for PVF/MMT 30% and PHB/MMT 20%, respectively. These findings showed that the adsorption rate relies on both the adsorbent membranes and the organic dyes.

## Figures and Tables

**Table 1:** The prepared samples’ compositions and spinning conditions.

	Composition (wt.%)			Electrospinning Parameters		
Sample Name	PHB	PVF	MMT	Flow Rate (mL·h^−1^)	Voltage (kV)	Distance to Collector (cm)
PVF	0	100	0	0.2	20	15
PVF/MMT 30%	0	70	30	0.5	20	15
PHB	100	0	0	0.5	20	20
PHB/MMT 10%	90	0	10	0.7	20	20
PHB/MMT 20%	80	0	20	0.5	20	20
PHB/PVF/MMT 10%	63	27	10	1.5	20	15

## Data Availability

Data are included in the manuscript.
